# Second victim syndrome in intensive care unit healthcare workers: A systematic review and meta-analysis on types, prevalence, risk factors, and recovery time

**DOI:** 10.1371/journal.pone.0292108

**Published:** 2023-10-03

**Authors:** Kazuaki Naya, Gen Aikawa, Akira Ouchi, Mitsuki Ikeda, Ayako Fukushima, Shuhei Yamada, Megumi Kamogawa, Shun Yoshihara, Hideaki Sakuramoto

**Affiliations:** 1 Department of Adult Health Nursing, Tokyo Healthcare University Wakayama Faculty of Nursing, Wakayama City, Wakayama, Japan; 2 Department of Adult Health Nursing, College of Nursing, Ibaraki Christian University, Hitachi, Ibaraki, Japan; 3 Department of Emergency and Critical Care Medicine, Graduate School of Comprehensive Human Sciences, University of Tsukuba, Tsukuba City, Ibaraki, Japan; 4 Department of Critical Care and Disaster Nursing, Japanese Red Cross Kyushu International College of Nursing, Munakata, Fukuoka, Japan; University of Ottawa, CANADA

## Abstract

**Introduction:**

Patient safety incidents, including medical errors and adverse events, frequently occur in intensive care units, leading to a significant psychological burden on healthcare workers. This burden results in second victim syndrome, which impacts the psychological and psychosomatic well-being of these workers. However, a systematic review focusing specifically on this condition among intensive care unit healthcare workers is lacking. Therefore, we aimed to conduct a systematic review and meta-analysis to examine the occurrence of second victim syndrome among intensive care unit healthcare workers, including the types, prevalence, risk factors, and recovery time associated with this condition.

**Methods:**

We conducted a comprehensive search of the MEDLINE, CINAHL, PsycINFO, and Igaku Chuo Zasshi databases. The eligibility criteria encompassed retrospective, prospective, and cross-sectional studies and controlled trials, with no language restrictions. Data on the type, prevalence, risk factors, and recovery time of second victim syndrome were extracted and pooled. Prevalence estimates from the included studies were combined using a random-effects meta-analytic model.

**Results:**

Of the 2,245 records retrieved, 16 potentially relevant studies were identified. Following full-text evaluation, five studies met the inclusion criteria and were included in the review. The findings revealed that 58% of intensive care unit healthcare workers experienced second victim syndrome. Frequent symptoms included guilt (12–68%), anxiety (38–63%), anger at self (25–58%), and lower self-confidence (7–58%). However, specific risk factors exclusive to intensive care unit healthcare workers were not identified in the review. Furthermore, approximately 20% of individuals took more than a year to recover or did not recover at all from the second victim syndrome.

**Conclusions:**

Thus, this condition is prevalent among intensive care unit healthcare workers and may persist for extended periods, potentially exceeding a year. The risk factors for second victim syndrome in the intensive care unit setting are unclear and require further investigation.

## Introduction

Patient safety incidents, including medical errors and adverse events, frequently occur in clinical practice and pose a threat to patients’ lives and well-being [1]. Individuals involved in these incidents, particularly healthcare workers, can also experience negative effects and are referred to as second victims [2, 3]. This phenomenon causes the second victim syndrome (SVS), characterized by psychological reactions such as anxiety and depression, as well as psychosomatic symptoms including headaches and sleep disturbances [2, 4].

Intensive care units (ICUs) are particularly susceptible to patient safety incidents, which can place a significant psychological burden on healthcare workers and lead to a high prevalence or exacerbation of SVS. In critically ill patients in the ICU, treatment is complex and high-risk, and the incidence of medical errors and adverse events is high, affecting patient outcomes [5–7]. Notably, studies have reported an 18% prevalence of SVS among ICU healthcare workers, highlighting its significance [8].

Despite the importance of understanding SVS in ICU healthcare workers, no systematic review has addressed this topic, to the best of our knowledge. Therefore, in this study, we aim to conduct a systematic review to explore SVS in ICU healthcare workers, including its types, prevalence, risk factors, and recovery time.

## Materials and methods

We conducted this systematic review and meta-analysis following the recommendations of the Joanna Briggs Institute Reviewer’s Manual [9] and the Preferred Reporting Items for Systematic Reviews and Meta-analyses Statement [10]. The systematic review protocol was registered with the International Prospective Register of Systematic Reviews (registration number: CRD42023389943).

### Eligibility criteria

In this study, SVS was defined as “negative impacts on health care workers, directly or indirectly involved in an unanticipated adverse patient event, unintentional healthcare error, or patient injury” [3]. We employed the context, condition, and population framework to guide the selection of eligible studies. The inclusion criteria were as follows: (1) context—healthcare workers exposed to patient safety incidents, including harmful incidents, near misses, and no-harm incidents, as defined by the Canadian Patient Safety Institute; (2) condition—second victim symptoms such as psychological responses and psychosomatic symptoms; (3) population—all ICU healthcare workers; and (4) study design—retrospective, prospective, and cross-sectional studies and controlled trials. No language restrictions were imposed, and studies for which full-text articles could not be obtained were excluded.

### Information sources and search strategy

We conducted searches in the MEDLINE (via PubMed), CINAHL (via EBSCOhost), PsycINFO (via Ovid), and Igaku Chuo Zasshi databases from inception to January 17, 2023. Ongoing trials were searched in the World Health Organization International Clinical Trials Registry Platform. The search terms used in all databases included “critical care,” “intensive care unit,” and “critical illness,” cross-referenced with the terms “medical errors,” “patient safety,” and “second victim syndrome” In addition, relevant studies were identified through hand searches of reference lists of the identified studies and articles (based on Google Scholar) that cited those studies from the period after the second screening to March 1, 2023. No language restrictions were applied. The complete search strategy used in all databases is presented in S1 Text.

### Selection process

Of the nine reviewers (KN, GA, AO, MI, AF, SY, MK, SY, and HS), two independently reviewed the titles and abstracts to identify potentially relevant studies. Two reviewers independently assessed eligibility based on full-text reviews. Disagreements between reviewers were resolved through consensus discussion or arbitration by a third reviewer.

### Data collection and outcome process

Data extraction included the following: author name, year of publication, study design, country, sample size, and characteristics of healthcare workers. Outcome data were extracted on the types, prevalence, and risk factors of SVS and recovery time from SVS. Data were documented using an Excel spreadsheet, and data extraction was performed independently by five reviewers (KN, GA, AO, AF, and HS). Authors of studies that did not provide details about the characteristics and outcomes of ICU healthcare workers were contacted to obtain data. To pool the results, data were extracted dichotomously from individual studies. The results were pooled by category.

### Assessment of study quality

The Mixed Methods Assessment Tool (MMAT) was used to evaluate the methodological quality of each study [11]. Studies were rated on a categorical scale as “no,” “can’t tell,” or “yes” to indicate whether they met the methodological quality criteria assessed. The number of items rated “yes” was counted to provide an overall score out of a possible 5, with a higher number corresponding to stronger methodological quality. Studies were appraised against the MMAT screening criteria by two researchers (KN and SY) independently. Instead of generating an overall score for each study, a qualitative approach was applied by providing a detailed review of study quality [11]. Studies were appraised as having a low, moderate, or high methodological quality.

### Statistical analysis

We pooled prevalence estimates from the included studies using a random-effects meta-analytic model. Heterogeneity across studies was assessed using I^2^ statistics. I^2^ was categorized as low (0–40%), moderate (30–60%), substantial (50–90%), or considerable (75–100%). The results are presented as forest plots with 95% confidence intervals (CIs). The analysis was conducted using R statistical software version 4.3.0 (R Development Core Team, 2008) and the “*meta”* package.

### Ethics

Ethical approval and patient consent were not required for this study.

## Results

### Selection and inclusion of studies

**Fig 1:**
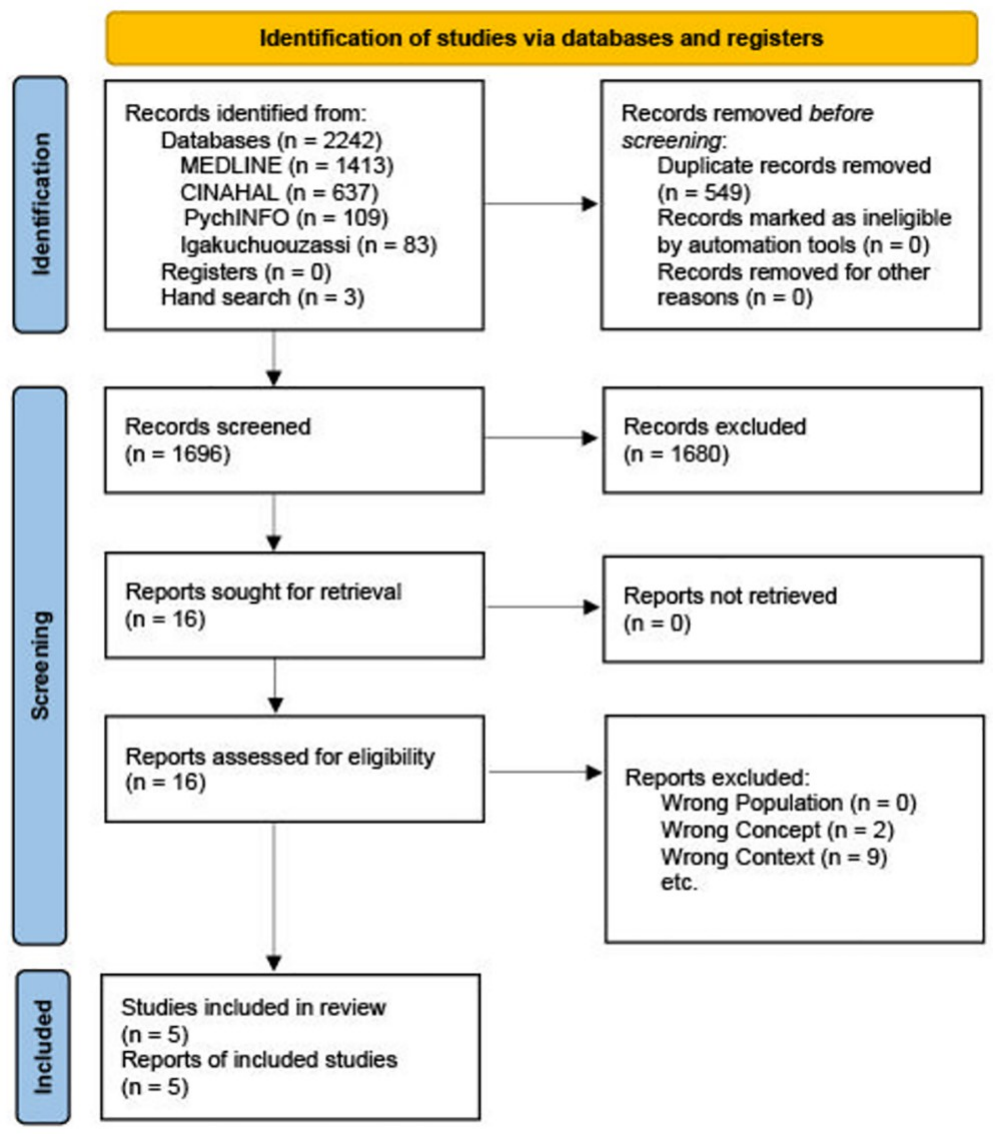
PRISMA 2020 flow diagram.

The electronic databases and hand search yielded 2,245 records (2,242 from electronic databases and 3 from hand search). After screening titles and/or abstracts, 16 full-text articles were assessed for eligibility. Eleven studies were subsequently excluded for various reasons such as being unrelated to the concept or context. Ultimately, five studies that met the inclusion criteria were included in the review (Fig 1).

The included articles were predominantly written in English and published between 2002 and 2023, with one study being in Portuguese [8]. One study was conducted in Brazil [8], another in the United States [12], and the remaining three in Germany [13–15]. The three German studies were conducted by the same research team.

All studies utilized a cross-sectional survey design. While some authors calculated only descriptive statistics, others additionally applied logistic regression models or chi-squared tests. All studies employed self-report questionnaires administered via paper-and-pencil or web-based/electronic platforms. Four studies were conducted with physicians or nurses [8, 13–15]. One study was conducted with a wide range of professionals, including physicians, nurses, respiratory therapists, and pharmacists from the Society of Critical Care Medicine [12]. The survey employed medical error scenarios to investigate the symptoms experienced by healthcare providers when faced with such situations (Table 1).

**Table 1:** Summarized findings of included articles.

Author	Country	Study design	Participants	Second victim symptoms		Recovery time	
Padilha et al., 2002 [8]	Brazil	Cross-sectional, questionnaire survey	148 ICU nurses from 7 hospitals	Anxiety	38%	None	
				Powerlessness	15%		
				Guilt	12%		
				Anger	10%		
				Worry	8%		
				Insecurity	7%		
				Despair	3%		
				Others	5%		
Kaur et al., 2019 [12]	United States	Cross-sectional, web-based questionnaire survey using medical error scenarios	148 clinicians working in ICUs who were members of the Society of Critical Care Medicine	Guilt	68%	None	
				Anxiety	63%		
				Anger at self	58%		
				Professional self-doubt	51%		
				Re-living the event over and over	48%		
				Fear of litigation	47%		
				Fear of judgment by colleagues	46%		
				Loss of sleep	44%		
				Shame	27%		
				Defensiveness	25%		
				Loss of concentration	24%		
				Depression	23%		
				Loss of reputation	18%		
				Anger at others	17%		
				Loss of interest in daily activities	14%		
				Considered career change	11%		
				Use of alcohol or other substances	5%		
				Thoughts of hurting oneself	3%		
				Did not experience any negative feelings after being involved in an adverse event	1%		
Strametz et al. [SeViD-I], 2021 [13]	Germany	Cross-sectional, web-based questionnaire survey	128 young (≤ 35 years) physicians working in ICUs and IMCs who were members of the German Society of Internal Medicine	Fear of social isolation from colleagues	30%	Less than one day	4%
				Fear of losing the job	19%	Within one week	29%
				Lethargy	34%	Within one month	40%
				Depressed mood	45%	Within one year	10%
				Concentration problems	36%	More than one year	1%
				Recall of the situation outside the workplace	45%	Never	10%
				Recall of the situation at the workplace	45%		
				Aggressive, risky behavior	9%		
				Defensive, overprotective behavior	44%		
				Psychosomatic reactions (headaches, back pain)	28%		
				Difficulties sleeping or excessive need to sleep	44%		
				Use of substances (alcohol/drugs) due to this event	22%		
				Feeling of shame	35%		
				Feeling of guilt	48%		
				Lower self-confidence	57%		
				Social isolation	18%		
				Anger against others	30%		
				Anger against oneself	35%		
				Desire to get support from others	52%		
				Desire to work through the incident for a deeper understanding	52%		
Strametz et al. [SeViD-II], 2021 [14]	Germany	Cross-sectional, web-based questionnaire survey	107 nurses working in ICUs and IMCs who were members of the German Society of Internal Medicine	Fear of social isolation from colleagues	17%	Less than one day	3%
				Fear of losing the job	16%	Within one week	29%
				Lethargy	30%	Within one month	20%
				Depressed mood	34%	Within one year	19%
				Concentration problems	36%	More than one year	2%
				Recall of the situation outside the workplace	25%	Never	15%
				Recall of the situation at the workplace	36%		
				Aggressive, risky behavior	9%		
				Defensive, overprotective behavior	36%		
				Psychosomatic reactions (headaches, back pain)	37%		
				Difficulties sleeping or excessive need to sleep	39%		
				Use of substances (alcohol/drugs) due to this event	13%		
				Feeling of shame	33%		
				Feeling of guilt	33%		
				Lower self-confidence	39%		
				Social isolation	12%		
				Anger against others	27%		
				Anger against oneself	25%		
				Desire to get support from others	37%		
				Desire to work through the incident for a deeper understanding	36%		
Marung et al. [SeViD-III], 2023 [15]	Germany	Cross-sectional, web-based questionnaire survey	184 physicians working at ICUs and IMCs who were members of the German Prehospital Emergency Physician Association	Fear of social isolation from colleagues	22%	Less than one day	2%
				Fear of losing the job	10%	Within one week	22%
				Lethargy	33%	Within one month	37%
				Depressed mood	38%	Within one year	20%
				Concentration problems	31%	More than one year	11%
				Recall of the situation outside the workplace	36%	Never	8%
				Recall of the situation at the workplace	38%		
				Aggressive, risky behavior	10%		
				Defensive, overprotective behavior	35%		
				Psychosomatic reactions (headaches, back pain)	18%		
				Difficulties sleeping or excessive need to sleep	35%		
				Use of substances (alcohol/drugs) due to this event	11%		
				Feeling of shame	27%		
				Feeling of guilt	40%		
				Lower self-confidence	44%		
				Social isolation	11%		
				Anger against others	21%		
				Anger against oneself	28%		
				Desire to get support from others	36%		
				Desire to work through the incident for a deeper understanding	42%		

ICU, intensive care unit; IMC, intermediate care unit; SeViD, Second Victims in Deutschland

The three studies conducted in Germany utilized the SeViD (Second Victims in Deutschland) questionnaire, which had been previously developed and validated by researchers [13–15]. These surveys were conducted among physicians and nurses of the German Society of Internal Medicine and the German Prehospital Emergency Physician Association.

### Quality assessment

Among the included studies, one study met 80% of the quality criteria, three met 60% of the quality criteria, and one met only 20% of the quality criteria. However, in most studies, the data were not considered representative of the general population because of sampling limitations in terms of specific regions or populations and low response rates (Table 2).

**Table 2:** Quality assessment of included studies using MMAT.

Types of mixed methods study components	Methodological quality criteria	Padilha et al. 2002 [8]	Kaur et al. 2019 [12]	Strametz et al. 2021 [13]	Strametz et al. 2021 [14]	Marung et al. 2023 [15]
4. Quantitative descriptive	4.1. Is the sampling strategy relevant to address the research question?	N	Y	Y	Y	Y
	4.2. Is the sample representative of the target population?	N	Y	N	N	N
	4.3. Are the measurements appropriate?	U	Y	Y	Y	Y
	4.4. Is the risk of nonresponse bias low?	Y	N	N	N	N
	4.5. Is the statistical analysis appropriate to answer the research question?	N	Y	Y	Y	Y
Score (%)		20	80	60	60	60

MMAT = Mixed Methods Appraisal Tool; Y = yes; N = no; U = cannot tell.

All studies that went through quality assessment passed the screening questions: 1) Are there clear research questions? 2) Do the collected data allow for addressing the research questions?

### Type and prevalence of second victim symptoms

The type and prevalence of each symptom experienced by the second victim are listed in Table 3. Symptoms were broadly categorized into thoughts, psychological distress, physical distress, impact on behavior, and impact on sociability. Frequent symptoms included guilt (12–68%), anxiety (38–63%), anger at self (25–58%), and lower self-confidence (7–57%).

**Table 3:** Prevalence of second victim symptoms.

Second victim symptoms	Prevalence	Reference
**Thought**		
Guilt	12–68%	Padilha et al. [8], Kaur et al. [12], Strametz et al. [13], Strametz et al. [14], Marung et al. [15]
Lower self-confidence	7–57%	Padilha et al. [8], Kaur et al. [12], Strametz et al. [13], Strametz et al. [14], Marung et al. [15]
Shame	27–35%	Kaur et al. [12], Strametz et al. [13], Strametz et al. [14], Marung et al. [15]
Worry	8%	Padilha et al. [8]
Despair	3%	Padilha et al. [8]
Fear		
of judgment by colleagues	17–46%	Kaur et al. [12], Strametz et al. [13], Strametz et al. [14], Marung et al. [15]
of losing the job	10–19%	Strametz et al. [13], Strametz et al. [14], Marung et al. [15]
of litigation	47%	Kaur et al. [12]
Anger	10%	Padilha et al. [8]
at self	25–58%	Kaur et al. [12], Strametz et al. [13], Strametz et al. [14], Marung et al. [15]
at others	17–30%	Kaur et al. [12], Strametz et al. [13], Strametz et al. [14], Marung et al. [15]
Thoughts of hurting oneself	3%	Kaur et al. [12]
Desire to work through the incident for a deeper understanding	36–52%	Strametz et al. [13], Strametz et al. [14], Marung et al. [15]
Desire to get support from others	36–52%	Strametz et al. [13], Strametz et al. [14], Marung et al. [15]
Considered career change	11%	Kaur et al. [12]
**Psychological distress**		
Anxiety	38–63%	Padilha et al. [8], Kaur et al. [12]
Depression	23–45%	Kaur et al. [12], Strametz et al. [13], Strametz et al. [14], Marung et al. [15]
Lethargy	15–34%	Padilha et al. [8], Strametz et al. [13], Strametz et al. [14], Marung et al. [15]
Re-living the event over and over	48%	Kaur et al. [12]
Recall of the situation at the workplace	36–45%	Strametz et al. [13], Strametz et al. [14], Marung et al. [15]
Recall of the situation outside the workplace	25–45%	Strametz et al. [13], Strametz et al. [14], Marung et al. [15]
**Physical distress**		
Loss of sleep	35–44%	Kaur et al. [12], Strametz et al. [13], Strametz et al. [14], Marung et al. [15]
Psychosomatic reactions (headaches, back pain)	18–37%	Strametz et al. [13], Strametz et al. [14], Marung et al. [15]
**Impacts on behavior**		
Concentration problems	24–36%	Kaur et al. [12], Strametz et al. [13], Strametz et al. [14], Marung et al. [15]
Defensive, overprotective behavior	25–44%	Kaur et al. [12], Strametz et al. [13], Strametz et al. [14], Marung et al. [15]
Aggressive, risky behavior	9–10%	Strametz et al. [13], Strametz et al. [14], Marung et al. [15]
Use of alcohol or other substances	5–22%	Kaur et al. [12], Strametz et al. [13], Strametz et al. [14], Marung et al. [15]
Loss of interest in daily activities	14%	Kaur et al. [12]
**Impacts on sociability**		
Loss of reputation	18%	Kaur et al. [12]
Social isolation	11–18%	Strametz et al. [13], Strametz et al. [14], Marung et al. [15]

### Risk factors of SVS and recovery time from SVS

In a survey conducted among clinician members of the Society of Critical Care Medicine [12], surgeons and anesthesiologists exhibited higher negative responses following procedural errors, whereas internal medicine and emergency medicine practitioners showed higher negative responses after diagnostic errors. Surveys conducted using the SeViD questionnaire did not exclusively identify risk factors for ICU healthcare workers.

The recovery time from SVS varied among ICU healthcare workers, with 2–4% recovering in less than one day, 22–29% within one week, 20–40% within one month, 10–20% within one year, 1–11% after more than one year, and 8–15% never recovering (Table 1).

### Meta-analysis of SVS prevalence

**Fig 2:**
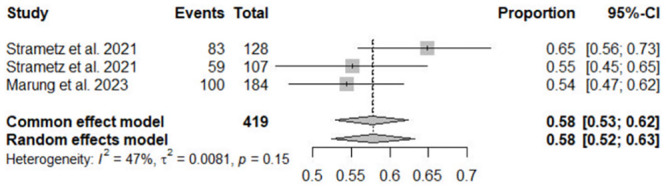
Forest plot for a lifetime prevalence of second victim syndrome CI, confidence interval.

**Fig 3:**
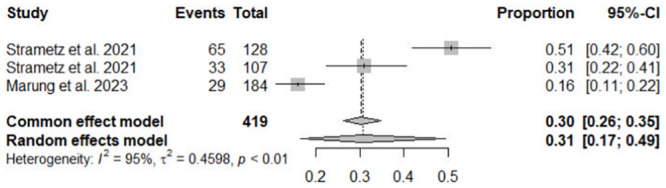
Forest plot for a 12-month prevalence of second victim syndrome CI, confidence interval.

Estimates of lifetime and 12-month prevalence of SVS were calculated in three studies [13–15]. The meta-analysis showed a lifetime prevalence of SVS at 58% (95% CI = 52–63, I^2^ = 47%) and a 12-month prevalence of SVS at 31% (95% CI = 17–49, I^2^ = 95%) (Figs 2 and 3). High heterogeneity was observed for the 12-month prevalence. However, subgroup- and nursing-specific analyses could not be conducted because of the limited number of articles.

One study was excluded from the meta-analysis because of its use of medical error scenarios and different methods compared to other studies [12]. Another study was excluded from the analysis because it did not calculate the incidence of SVS [8].

## Discussion

Our study demonstrated that the majority of ICU healthcare workers experienced SVS, most lasting for more than one year. The risk factors for SVS in ICU healthcare workers remain unclear.

To the best of our knowledge, this is the first systematic review to focus on SVS among ICU healthcare workers. Our study showed that 58% of ICU healthcare workers had SVS, which is higher than the incidence (10–43%) reported for healthcare workers in various healthcare settings (e.g., operating rooms, obstetrics, and internal medicine) [16]. This could be because ICU healthcare workers frequently encounter patient safety incidents, and their psychological impact is significant. The ICU targets critically ill patients and provides highly invasive treatment using many complex medical devices. The frequency of medical procedures is also high, which inevitably leads to patient safety incidents such as adverse events and medical errors. Between 20% and 51% of critically ill patients experience an adverse event during their ICU stay, which is higher than an incidence of 10% in healthcare overall [6, 17, 18]. The incidence of preventable adverse events is also 18% in ICUs, higher than that in general wards [19]. These adverse events are associated with longer ICU and hospital stays [7]. Furthermore, the incidence of human error in the ICU is 31%, leading to fatal or permanent injury or prolonging the length of ICU stay [5]. Therefore, more ICU healthcare workers may be involved in patient safety incidents, and the impact of these incidents on patients may be a major psychological burden for healthcare workers.

Our findings showed that the symptoms experienced by second victims in the ICU, such as guilt, anxiety, lower self-confidence, and re-living the event repeatedly, were similar to those reported in other systematic reviews on SVS for healthcare workers [16]. A previous meta-analysis of all healthcare workers showed that “troubling memories” (81%, 95% CI = 46–95), “anxiety/concern” (76%, 95% CI = 33–95), and “anger toward themselves” (75%, 95% CI = 59–86) were the most frequent symptoms among second victims [20]. Both ICU healthcare workers and healthcare workers in general tend to have distressing memories associated with patient safety events. Unwanted, upsetting memories and flashbacks are common after traumatic experiences [21, 22]. Therefore, focusing on the presence or absence of these symptoms would be necessary.

Approximately 60% of individuals who experienced SVS recovered within one month, while approximately 20% took more than a year to recover or did not recover at all. The impact of prolonged SVS is unknown owing to lack of evidence. However, as SVS is associated with turnover intention and absenteeism, addressing patient safety events early on is necessary for healthcare providers [23]. For example, support programs such as peer support and learning from adverse events are desired by second victims, and their availability and effectiveness have been reported [24–26].

Despite the significant findings, we acknowledge certain limitations of our study. First, the meta-analysis of 12-month SVS prevalence showed high heterogeneity, which may be attributable to the variability in the probability of encountering a patient safety event within one year. The heterogeneity of SVS prevalence over a lifetime is lower than this, implying that the differences in prevalence by occupation may not be large. Second, the generalizability of the findings is limited because of the scarcity of studies on SVS in ICUs and the analysis being conducted on data from a single country. This is because mental health symptoms such as anxiety and depression differ between countries and regions [27]. Furthermore, these symptoms can be influenced by the organization’s patient safety culture and the health of the work environment [28]. Third, the three SeViD studies integrated in the meta-analysis were categorized as involving healthcare professionals affiliated with ICUs or intermediate care units; thus, the prevalence of SVS may not strictly involve healthcare workers only in ICUs [13–15]. In addition, the studies included in this research were mainly limited to physicians or nurses; therapists, pharmacists, engineers, and other professionals working in ICUs were not studied, and the prevalence of SVS among them is unknown.

The high prevalence of SVS among ICU healthcare workers and its potential for prolonged impact necessitate follow-up and support. However, research on SVS in ICUs is limited, and the prevalence by professional category, risk factors, prolonged impact, and desired support remain unclear. Therefore, future research investigating the prevalence of SVS by professional category, risk factors, and its prolonged impact as well as identifying the desired support resources in ICUs can provide basic data for preventing and reducing SVS in ICUs. A step-by-step approach is recommended for the desired support resources, including the help of colleagues in the department, crisis intervention by a special team, and a support network of professionals [29]. Verifying the effects of these resources in ICUs will be necessary in the future.

Further research on SVS is warranted, but caution should be exercised when investigating it. This is because it is particularly similar to concepts such as moral distress and burnout, and a distinction must be made between them. A second victim has been defined as “Any health care worker, directly or indirectly involved in an unanticipated adverse patient event, unintentional healthcare error, or patient injury, and who becomes victimized in the sense that they are also negatively impacted” by experts in a recent article [3]. Research on SVS would need to be conducted based on this definition.

## Conclusions

This systematic review highlighted that SVS is a frequently prevalent issue among ICU healthcare workers, especially with symptoms such as guilt, anxiety, lower self-confidence, and re-living the event. These symptoms can have a prolonged impact, lasting for more than a year. The findings of this study emphasized the importance of addressing SVS and providing appropriate support for ICU healthcare workers who experience patient safety incidents. Early interventions and support programs tailored to the unique needs of ICU professionals are crucial for their well-being and to mitigate the potential negative consequences of SVS. However, the risk factors for SVS in the ICU setting remain unclear and require further investigation. By gaining a deeper understanding of the factors contributing to SVS and implementing targeted interventions, healthcare organizations can create a safer and more supportive environment for their frontline staff, ultimately enhancing patient safety and well-being.

## Supporting information

S1 Text Search terms. (DOCX)

S1 Checklist PRISMA 2020 checklist. (DOCX)
