# Supplementary material for: Second victim syndrome in intensive care unit healthcare workers: A systematic review and meta-analysis on types, prevalence, risk factors, and recovery time
Source: PLoS One. 2023 Oct 3;18(10):e0292108. doi: 10.1371/journal.pone.0292108 (PMC10547210; doi:10.1371/journal.pone.0292108)
Supplement: S1 Text — (DOCX) [file pone.0292108.s001.docx]

**S1 Text**

**Search Terms**

**PubMed**

Critical care[mh] OR Intensive care units[mh] OR Critical illness[mh] OR "critical care"[tiab] OR "intensive care unit"[tiab] OR "intensive care"[tiab] OR ICU[tiab] OR "critical illness"[tiab]

OR "critically ill"[tiab]

AND

Health Personnel[mh] OR "medical staff"[tiab] OR "health care provider"[tiab] OR "health personnel"[tiab] OR "Healthcare Worker"[tiab] OR "healthcare professional*"[tiab] OR "midwi*"[tiab] OR "nurse"[tiab] OR "physician"[tiab] OR "clinician"[tiab]

AND

Medical Errors[mh] OR Malpractice[mh] OR Patient Safety[mh] OR Risk Management[mh] OR "medical error"[tiab] OR "medication Error"[tiab] OR "diagnostic error"[tiab] OR "medical mistake*"[tiab] OR "administration error"[tiab] OR "medical incident*"[tiab] OR "patient safety incident"[tiab] OR "nursing error"[tiab] OR "human error"[tiab] OR "adverse event*"[tiab] OR "near miss"[tiab] OR "Risk Management"[tiab] OR "Malpractice"[tiab] OR "Patient Safety"[tiab]

OR

Psychology [mh] OR Emotions[mh] OR Mental Health[mh] OR Anxiety[mh] OR Depression[mh] OR Depressive Disorder[mh] OR Stress Disorders, Traumatic[mh] OR

Mental Health Recovery[mh] OR Burnout, Psychological[mh] OR "second victim*"[tiab] OR self-esteem[tiab] OR "psychology "[tiab] OR "feeling*"[tiab] OR "emotion*"[tiab] OR "mental health"[tiab] OR "guilt"[tiab] OR "anxiet*"[tiab] OR "depress*"[tiab] OR "depressive disorder*"[tiab] OR PTSD[tiab] OR "post-traumatic stress disorder"[tiab] OR "recover*"[tiab] OR "Mental Health Recovery"[tiab] OR "psychological response*"[tiab] OR "psychological symptom*"[tiab] OR "psychological impact"[tiab] OR "emotional response*"[tiab] OR "emotional reaction*"[tiab] OR "distress"[tiab] OR "burnout"[tiab] OR "clinical-judicial syndrome"[tiab] OR "repetitive negative thinking"[tiab] OR "avoidance behavior*"[tiab] OR

"well-being"[tiab] OR "self-efficacy"[tiab] OR "self-confidence"[tiab]

OR

"psychosomatic symptom*"[tiab] OR "sleep disturbance*"[tiab] OR "gastrointestinal symptom*"[tiab] OR "cardiovascular symptom*"[tiab] OR

NOT

animals [mh] NOT humans [mh]

**CINAHL via EBSCOhost**

(MH critical care) OR (MH Intensive care units) OR (MH Critical illness) OR (“critical care”) OR ( “intensive care unit”) OR (“intensive care”) OR (ICU) OR (“critical illness”) OR (“criticaliy ill”)

AND

(MH Health Personnel) OR (“medical staff”) OR (“health care provider”) OR (“halth personnel”) OR (“Healthcare Worker”) OR (“health professional”) OR (“midiwi”) OR (“nurse”) OR (“physician”) OR (“clinician”) OR ("staff")

AND

(MH Malpractice) OR (MH Patient Safety)OR (MH Risk Management) OR ("medical error*") OR ("medication Error") OR ("diagnostic error") OR ("medical mistake") OR ("administration error") OR ("medical incident") OR ("patient safety insident") OR ("nursing error") OR ("human error") OR ("adverse event*") OR ("near miss") OR ("Risk Management") OR ("Malpractice") OR ("Patient Safety")

AND

(MH Psychology) OR (MH Emotions) OR (MH Mental Healht) OR (MH Anxiety) OR (MH Depression) OR ("second victim*") OR (self-esteem) OR ("psychology") ("feeling*") OR ("emotion") OR ("mental health") OR ("guilt") OR ("anxiet*") OR ("depress*") OR ("depressive disorder*") OR (PTSD) OR ("post-traumatic stress disorder) OR ("recover*") OR ("Mental Health Recovery") OR ("psychological response*") OR ("psychological symptom*") OR ("psychological impact") OR ("emotional response*") OR ("emotional reaction") OR ("distress") OR ("burnout") OR ("repetitive negative thinking") OR ("adoidance behavio*") OR ("well-being") OR ("self-efficasy") OR ("self-confidence")

OR

("psychosomatic symptom*") OR ("sleep disturbance*") OR ("gastrointestinal symptom*") OR ("cardiovascular symptom*")

NOT

(MH animals) NOT (MH humans)

**PsycINFO**

exp intensive care/ OR "critical care".ab,ti. OR "intensive care unit".ab,ti. OR "intensive care".ab,ti. OR ICU.ab,ti. OR "critical illness".ab,ti. OR "critically ill".ab,ti.

AND

exp health personnel/ OR "medical staff".ab,ti. OR "health care provider".ab,ti. OR "health personnel".ab,ti. OR "healthcare worker".ab,ti. OR "healthcare professional*".ab,ti. OR "midwi*".ab,ti. OR "nurse".ab,ti. OR "physician".ab,ti. OR "clinician".ab,ti.

AND

exp professional liability/ OR exp patient safety/ OR exp risk management/ OR "medical error".ab,ti. OR "medication Error".ab,ti. OR "diagnostic error".ab,ti. OR "medical mistake*".ab,ti. OR "administration error".ab,ti. OR "medical incident*".ab,ti. OR "patient safety incident".ab,ti. OR "nursing error".ab,ti. OR "human error".ab,ti. OR "adverse event*".ab,ti. OR "near miss".ab,ti. OR "risk management".ab,ti. OR "malpractice".ab,ti. OR "patient safety".ab,ti.

AND

exp attitudes/ OR exp psychology/ OR exp emotions/ OR exp empathy/ OR exp mental health/ OR exp mental health/ OR exp depression/ OR exp major depression/ OR exp "stress and trauma related disorders"/ OR exp occupational stress/ OR "second victim*".ab,ti. OR self-esteem.ab,ti. OR "attitude*".ab,ti. OR "psychology ".ab,ti. OR "feeling*".ab,ti. OR "emotion*".ab,ti. OR "mental health".ab,ti. OR "guilt".ab,ti. OR "anxiet*".ab,ti. OR "depress*".ab,ti. OR "depressive disorder*".ab,ti. OR PTSD.ab,ti. OR "post-traumatic stress disorder".ab,ti. OR "concentrat*".ab,ti. OR "recover*".ab,ti. OR "mental health recovery".ab,ti. OR "perception*".ab,ti. OR "psychological response*".ab,ti. OR "psychological symptom*".ab,ti. OR "psychological impact*".ab,ti. OR "emotional response*".ab,ti. OR "emotional reaction*".ab,ti. OR "distress".ab,ti. OR "burnout".ab,ti. OR "clinical-judicial syndrome".ab,ti. OR "memor*".ab,ti. OR "repetitive negative thinking".ab,ti. OR "avoidance behavior*".ab,ti. OR "well-being".ab,ti. OR "self-efficacy".ab,ti. OR "self-confidence".ab,ti.

AND

"psychosomatic symptom*".ab,ti. OR "sleep disturbance*".ab,ti. OR "gastrointestinal symptom*".ab,ti. OR "cardiovascular symptom*".ab,ti.

**Igaku Chuo Zasshi**

クリティカルケア/TH OR ICU/TH OR 危篤/TH OR クリティカルケア/TA OR ICU/TA OR 危篤/TA OR 重病/TA OR 重症/TA

AND

医療周辺従事者/TH OR 保健医療従事者/TH OR 医療周辺従事者/TA OR 保健医療従事者/TA OR 医療専門職/TA OR 助産師/TA OR 看護職管理者/TH OR 看護師/TA OR 医師/TA

AND

医療ミス/TH OR 医療過誤/TH OR 患者の安全/TH OR リスクマネジメント/TH OR 医療ミス/TA OR 投薬ミス/TA OR 誤診/TA OR 医療事故/TA OR 患者安全事故/TA OR 看護ミス/TA OR ヒューマンエラー/TA OR 医薬品有害事象/TA OR ニアミス事例/TA OR インシデント・レポート/TH OR 患者の安全/TA

AND

態度/TH OR 心理学/TH OR 感情/TH OR 共感/TH OR 精神保健/TH OR 不安/TH OR 抑うつ/TH OR うつ病/TH OR ストレス障害-心的外傷性/TH OR 精神障害からの回復/TH OR バーンアウト/TH OR 第二の被害者/TA OR 自尊心/TA OR 態度/TA OR 心理学/TA OR 感情/TA OR 共感/TA OR 精神保健/TA OR 罪悪感/TA OR 不安/TA OR 抑うつ/TA OR うつ病/TA OR ストレス障害-心的外傷性/TA OR 注意（心理学）/TA OR 精神障害からの回復/TA OR experience/TA OR 知覚/TA OR 心理的反応/TA OR 行動心理学的徴候/TA OR 心理的影響/TA OR 情動反応/TA OR 苦痛/TA OR 記憶/TA OR バーンアウト/TA OR 回避学習/TA OR 自己効力感/TA OR 自信/TA

AND

リアリティーショック/TA OR 心身症/TA OR 睡眠-覚醒障害/TA OR 消化器症状/TA OR 循環器症状/TA

NOT

動物/TH NOT ヒト/TH
